# First Record of the Myrmicine Ant Genus *Meranoplus* Smith, 1853 (Hymenoptera: Formicidae) from the Arabian Peninsula with Description of a New Species and Notes on the Zoogeography of Southwestern Kingdom Saudi Arabia

**DOI:** 10.1371/journal.pone.0111298

**Published:** 2014-11-06

**Authors:** Mostafa R. Sharaf, Hathal M. Al Dhafer, Abdulrahman S. Aldawood

**Affiliations:** Plant Protection Department, College of Food and Agriculture Science, King Saud University, Riyadh, Saudi Arabia; University of Minnesota, United States of America

## Abstract

The ant genus *Meranoplus* is reported for the first time from the Arabian Peninsula (Kingdom of Saudi Arabia) by the new species *M. pulcher* sp. n., based on the worker caste. Specimens were collected from Al Sarawat and Asir Mountains of southwestern Kingdom of Saudi Arabia using pitfall traps. *Meranoplus pulcher* sp. n. is included in the Afrotropical *M. magretii*-group, with greatest similarity to *M. magrettii* André from Sudan. A key to the Afrotropical species of the *M. magretii*-group is presented. A brief review of the ant taxa with Afrotropical affinities in southwestern region of the Kingdom of Saudi Arabia is given.

## Introduction

Since Smith [1] established the genus *Meranoplus*, 87 species and subspecies are currently included [2]. This genus is broadly distributed in the Old World tropics, ranging through the Afrotropical, Malagasy, Oriental, and Indo-Australian regions [3]–[5]. The genus is absent from the Palaearctic and Oceania regions, except for a single species, *M. levellei* Emery, 1883 from New Caledonia [6], [7].

*Meranoplus* has been historically placed in the tribe Meranoplini, which also includes the genera *Dicroaspis* Emery, 1908 and *Calyptomyrmex* Emery, 1887 [8]. Recently, the tribe Meranoplini was restricted to two genera, *Meranoplus* [9] and a fossil genus *Parameranoplus* Wheeler, 1915 [10]. The *Meranoplus* fauna has been revised for most zoogeographical realms including the Afrotropical [3], Oriental [11], [12], Australian [13]–[17], and Malagasy [5]. Among the previous regions, the Australian region is the most taxa rich in regard to the number of species [3], [13].

Available information on the habitats and biology of *Meranoplus* species is limited. Most species nest directly in the soil, under stones, in rotten wood or in leaf litter [3], [4], [13]. African species have been reported to nest either in the ground with a crater type of entrance, or at the base of plants. Nests of one or two species are constructed among roots with workers ascending trees or low shrubs [3]. The majority of species are generalized omnivores [13], whereas others are considered seed harvesters, *e. g. M. diversus*-group [4], [13], [18].

The specialized morphology of the genus *Meranoplus* is related to a specialized behaviors, thanatosis, “playing dead” and and becoming cryptic. Several species when disturbed, fold their legs beneath the promesonotal shield and quickly accumulate dirt on body hairs and remain motionless in a fetal-like position [19], [20].

A thorough diagnosis of the genus *Meranoplus* has been provided by Bolton [3], [21]. The genus can be recognized by the combination of the following traits, masticatory margin of mandibles armed with four to five teeth; palp formula 5, 3; clypeus large, with median portion usually carinate at each side; antennal scrobes well-developed, usually long; antennae nine-segmented with a three segmented club; eyes large, located behind midlength of head, sometimes close to the posterior corners of head; pronotum and mesonotum fused into a plate or shield that extends posteriorly and laterally so the mesosomal sides, and usually also the propodeum, are concealed in dorsal view; lateral and/or posterior margins of promesonotal shield usually armed with spines, lobes, foliacious processes; petiole sessile usually cuneate in lateral view.

The *M. magrettii*-group can be distinguished by the following characters [3]: masticatory margin of mandibles armed with four or five teeth; mesosoma when seen from above with the propodeum concealed by the broad promesonotal shield; propodeal spines present; petiole cuneate in lateral view with unarmed dorsal surface; postpetiole broad and nodiform.

The *M. magrettii*-group has only two species known from the Afrotropical region, *M. magrettii* a species that is broadly distributed in the region and inhabiting savannah, grassland and dry woodland, and *M. peringueyi* apparently confined to South Africa [3].

The southwestern mountainous region of the Kingdom of Saudi Arabia (KSA) is one of the most diverse in terms of species diversity and relative abundances of insects. Taxonomic and biogeographical studies have indicated that the insects of this region have strong affinities with the Afrotropical Region [21]–[33]. A recent and comprehensive study of the Afrotropical relationships of the region is by El-Hawagryi *et al.* [32]. These authors recorded 17 orders of insects representing 129 families and at least 582 species and subspecies from Al-Baha Province. Their biogeographic analysis of the species composition clearly revealed that this region has a close affinity with the insect fauna of the Afrotropical Region.

In the present study, the myrmicine ant genus *Meranoplus* is recorded for the first time from KSA by the new species *M. pulcher* sp. n. and represents a new generic record for the Arabian Peninsula. In addition, a synopsis of the similarity of the Afrotropical fauna with the southwestern region of KSA is presented based on available records of ants.

## Materials and Methods

### Study area

Shada Al Ala Mountain is a parallel extension of Hijaz Mountains to the west. This region is managed as a natural protectorate in southwestern of Al Baha Province, 20 km northwest of Al Mukhwah Governorate. The region has a substantial high diversity of wild plants including *Albizia lebbeck* (L.) Benth. (Fabaceae), *Solenostemon* Thonn.(Lamiaceae), *Juniperus procera* Hochst. ex Endlicher (Cupressaceae), *Santalum* L. (Santalaceae), *Pimpinella anisum* L. (Apiaceae), *Rhamnus frangula* L. (Rhamnaceae), *Opuntia ficus-indica* (L.) Mill. (Cactaceae), *Ricinus communis* L. (Euphorbiaceae), *Olea europaea* ssp. *africana* (Mill.) P. Green. (Oleaceae), *Prunus dulcisn* (Mill.) D.A.Webb (Rosaceae), *Maerua crassifolia* Forssk. (Capparceae), *Pandanus tectorius* Parkinson (Pandanaceae), *Panicum Turgidum* Forssk. *(*Poaceae), *Coffea arabica* L. (Rubiaceae), *Opuntia ficus-indica* (L.) Mill. (Cactaceae), *Breonadia salicina* (Vahl) Hepper & J.R.I.Wood (Rubiaceae), *Haloxylon salicornicum* (Moq.) Bunge ex Boiss. (Chenopdiaceae), *Lycium shawii* Roem. & Schult (Solanaceae), *Cactus* (Cactaceae), and *Acacia* spp. (Memosaceae).

### Sampling procedures

Materials listed in this work, the holotype and 29 paratype specimens were collected during an insect inventory of the southwestern region of KSA by using more than 900 pitfall traps. A single specimen of the new species was collected by an aspirator from a *Cactus* sp. All the materials is deposited in King Saud University Museum of Arthropods, College of Food and Agriculture Sciences (KSMA), King Saud University, Riyadh, Kingdom of Saudi Arabia, except a single paratype specimen in California Academy of Sciences (CASC), San Francisco, USA.

All measurements and indices are expressed in millimeters and follow the standards of Boudinot & Fisher [5].

### Measurements

ATL: *Abdominal Tergum IV Length*. Maximum length of fourth abdominal tergum measured with anterior and posterior margins in same plane of focus.

ATW: *Abdominal Tergum IV Width*. Maximum width of fourth abdominal tergum with anterior, posterior, and lateral borders in same plane of focus.

CDD: *Clypeal Denticle Distance*. Distance between clypeal denticle apices, measured in full-face view.

CW: *Clypeus Width*. Distance between the apices of the frontal lobes across the clypeus.

EL: *Eye Length*. Maximum eye length in profile view.

EW: *Eye Width*. Maximum eye width in profile view.

HL: *Head Length*. Maximum length of head capsule, excluding mandibles, measured from anterior margin of clypeus to nuchal carina, with both in same plane of focus.

HLA: *Head Length, Anterior*. Distance between the anterior edges of the eyes to the mandible bases in full-face view.

HW: *Head Width*. Maximum width of head capsule behind the eyes, in full-face view.

PML: *Promesonotum Length*. Maximum length of promesonotum from posterior spine/denticle apices to anterolateral denticle apices; all four apices in same plane of focus. ( =  PMD, [15])

PPH: *Postpetiole Height*. Measured from sternal process base to postpetiole apex in lateral view.

PPL: *Postpetiole Length*. Measured from anterior to posterior inflections of postpetiole node in lateral view.

PWA: *Promesonotal Width, Anterior*. Maximum width of promesonotal shield between anterolateral denticle apices in dorsal view. ( =  PW, [15])

PWP: *Promesonotal Width, Posterior.* Distance between posterior-most promesonotal spine or denticle apices.

PTH: *Petiole Height*. Measured from petiole sternum to apex in lateral view.

PTL: *Petiole Length*. Measured from anterior to posterior inflections of petiole node.

SL: *Scape Length*. Maximum length of the scape excluding basal constriction.

SPL: *Propodeal Spine Length*. Workers: distance from inner posterior margin of propodeal spiracle to propodeal spine apex. Gynes: maximum propodeal spine length from basal inflection of spine, to spine apex.

WL: *Weber's Length*. Maximum diagonal length of mesosoma from anterior inflection of pronotum to posterolateral corner of the metapleuron or the metapleural lobes, whichever is most distant.

### Indices

CDI: *Clypeal Denticle Index*. CDD×100/CML

CI: *Cephalic Index*. HW×100/HL

CS: *Cephalic Size*. (HW+HL)/2

EYE: *Eye Index*. 100× (EL+EW)/CS

OMI: *Ocular-Mandibular Index*. EL×100/HLA

PMI: *Promesonotum Index 1*. PWA×100/PML ( =  PMI2, [15])

PPI: *Postpetiole Index*. PPL×100/PPH

PTI: *Petiole Index*. PTL×100/PTH

PWI: *Promesonotum Index 2*. PWP×100/PML

SEI: *Scape-Eye Index*. EL×100/SL

SI: *Scape Index*. SL×100/HW

### Illustrations

Specimens were photographed by Michele Esposito (CASC) using a JVC KYF70B3CCD digital camera attached to a Leica M420 stereomicroscope. All digital images were processed using Auto-Montage (Syncroscopy, Division of Synoptics Ltd, USA) software. Images of the specimens are available in full color on www.antweb.org. The map was created by the ArcGIS 9.2 program, with the help of Prof. Mahmoud S. Abdel-Dayem (King Saud University).Specimens were examined and imaged using scanning electron microscope (SEM) (JSM-6380 LA) visualization to record morphological details of the new species. The JSM-6380 LA is a high-performance scanning electron microscope with a high resolution of 3.0 nm.

No specific permits were required for the described field studies or for the surveyed locations which are not privately-owned or protected in any way or do not have endangered or protected species.

### Nomenclatural acts

The electronic edition of this article conforms to the requirements of the amended International Code of Zoological Nomenclature, and hence the new names contained herein are available under that Code from the electronic edition of this article. This published work and the nomenclatural acts it contains have been registered in ZooBank, the online registration system for the ICZN. The ZooBank LSIDs (Life Science Identifiers) can be resolved and the associated information viewed through any standard web browser by appending the LSID to the prefix "http://zoobank.org/". The LSID for this publication is: urn:lsid:zoobank.org:pub:80A78374-53CF-4A19-90CB-4A392E626999. The electronic edition of this work was published in a journal with an ISSN, and has been archived and is available from the following digital repositories: PubMed Central, LOCKSS.

## Results

### *Meranoplus pulcher* SHARAF sp. n. (Figs. 1–11)

**Figure 1:**
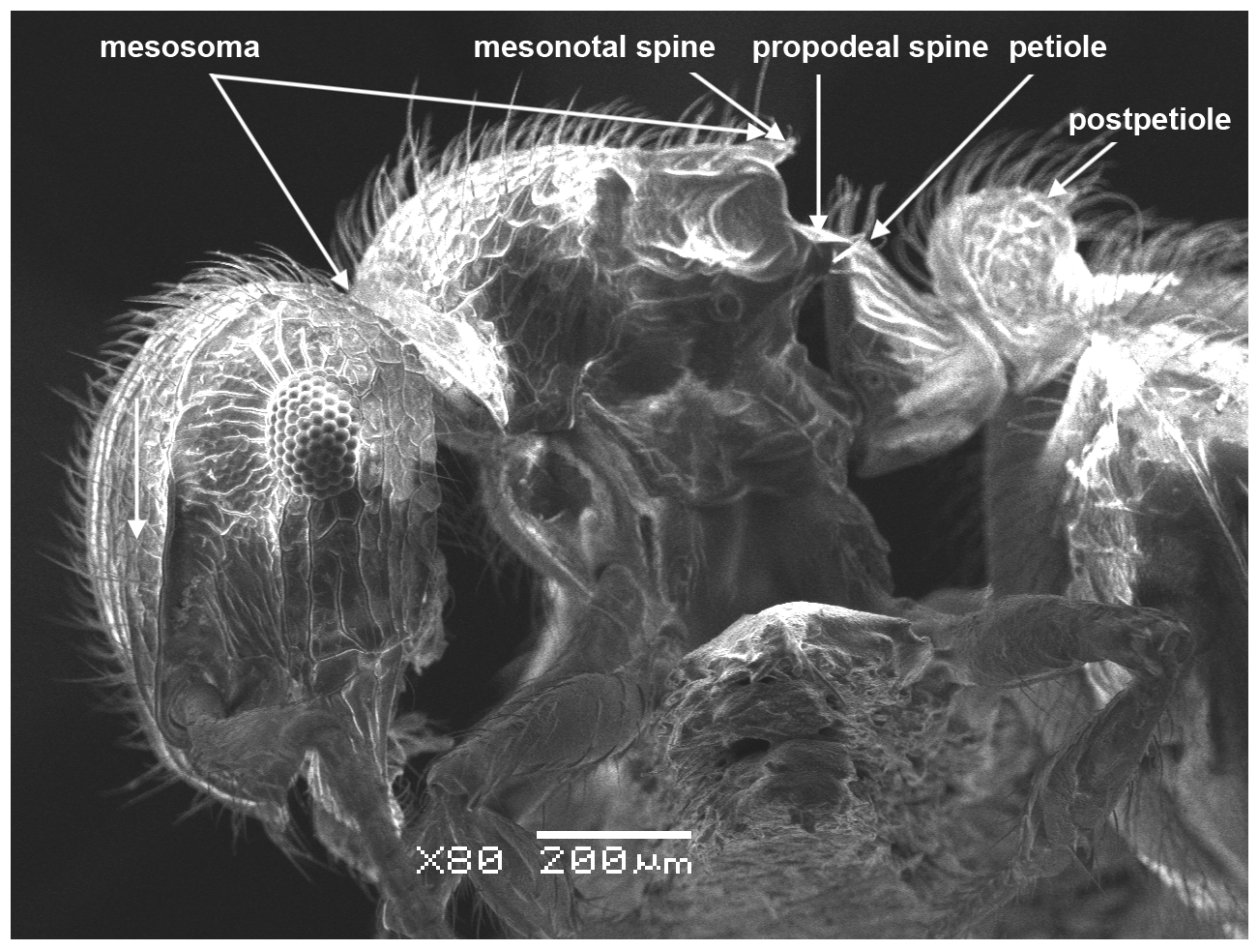
*Meranoplus pulcher* sp. n., paratype worker, body in profile.

**Figure 2:**
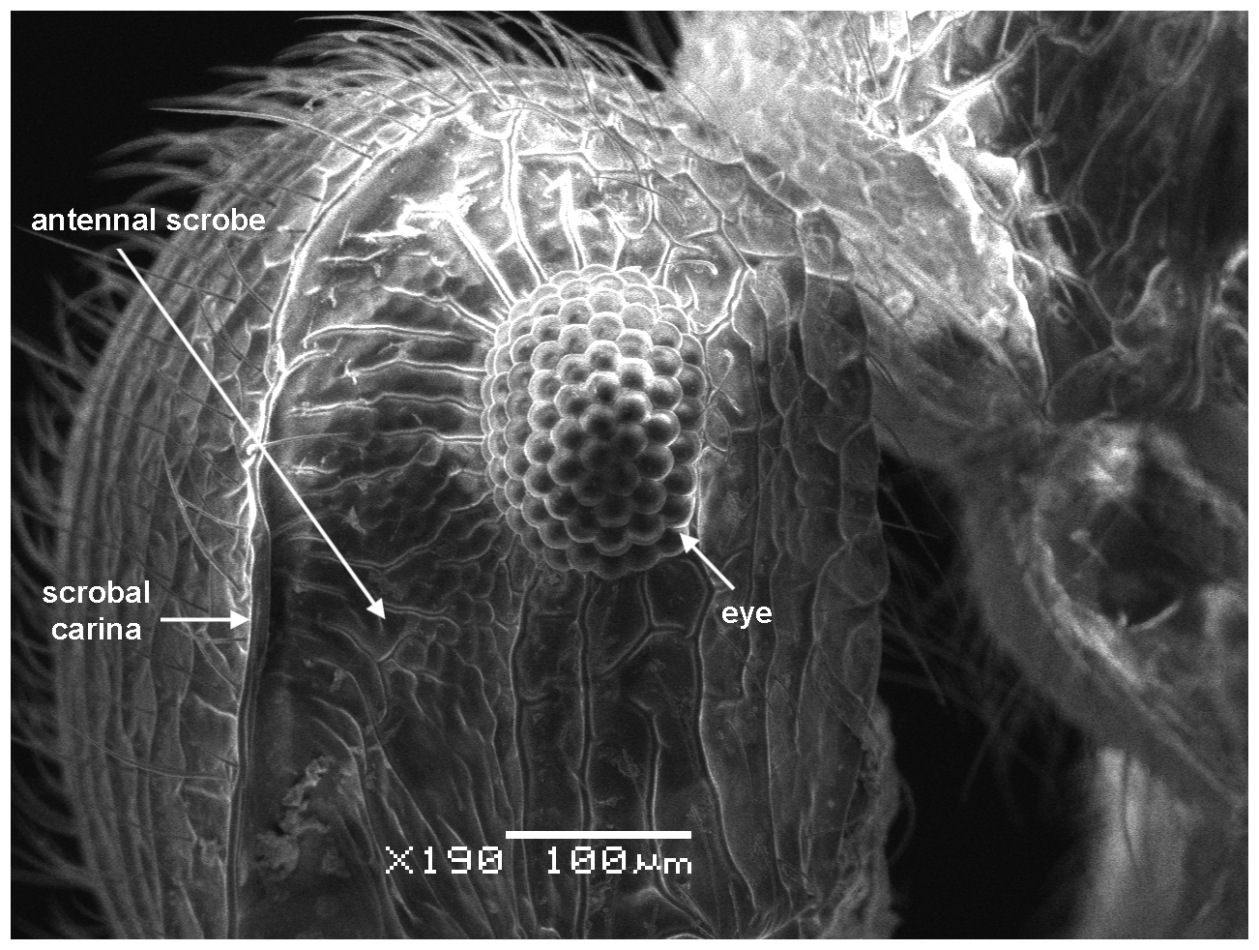
Head in profile.

**Figure 3:**
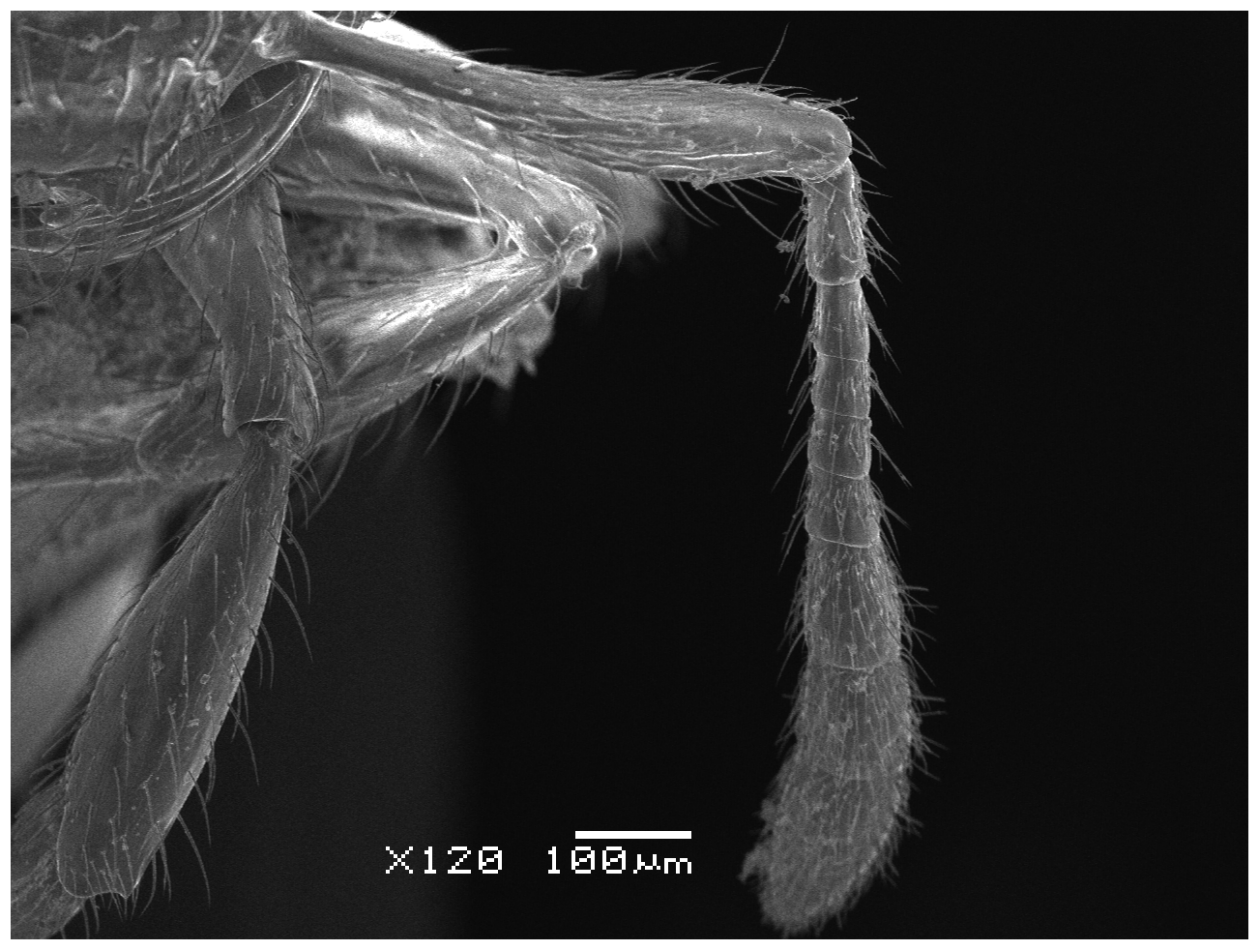
Antenna.

**Figure 4:**
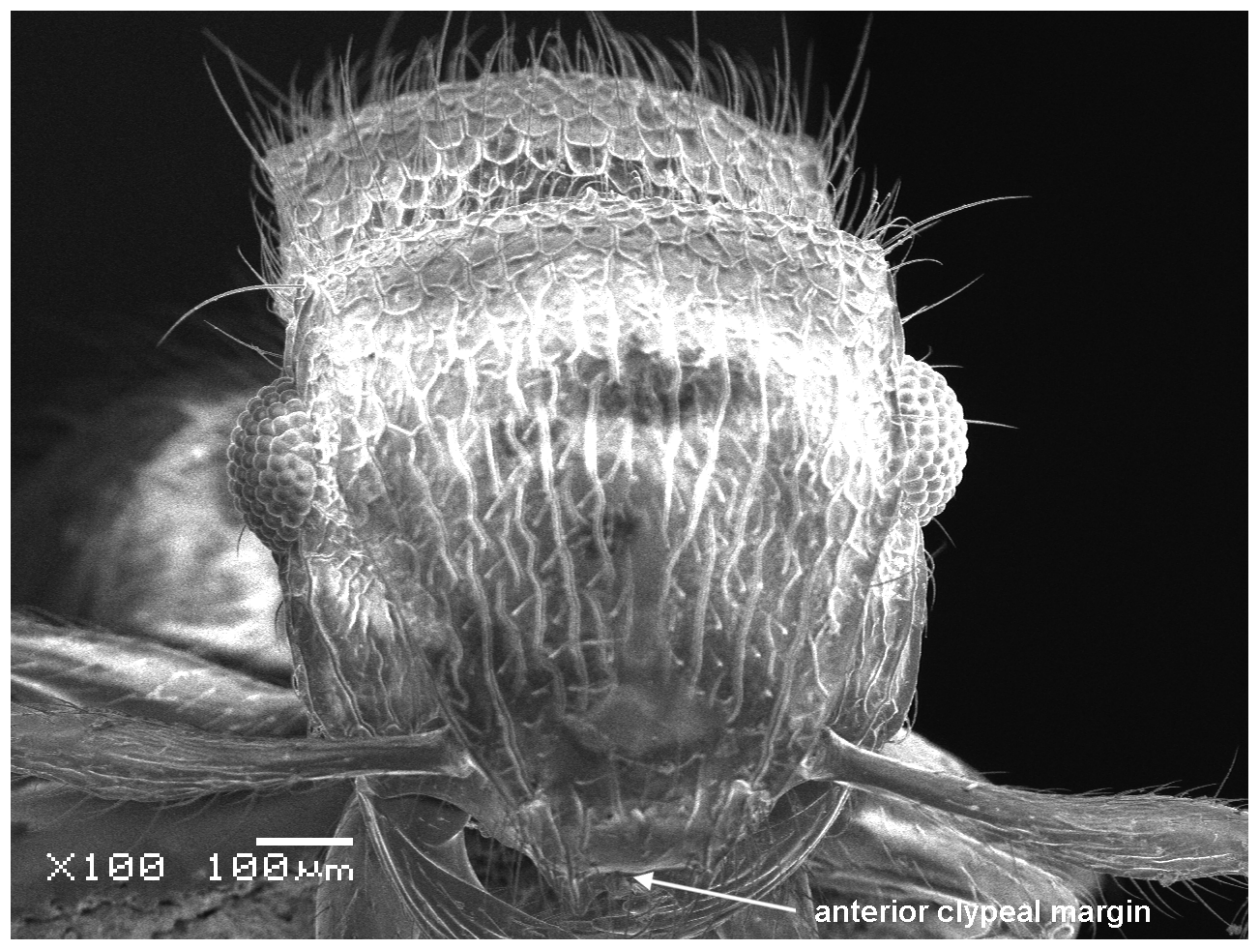
Head in full-face view.

**Figure 5:**
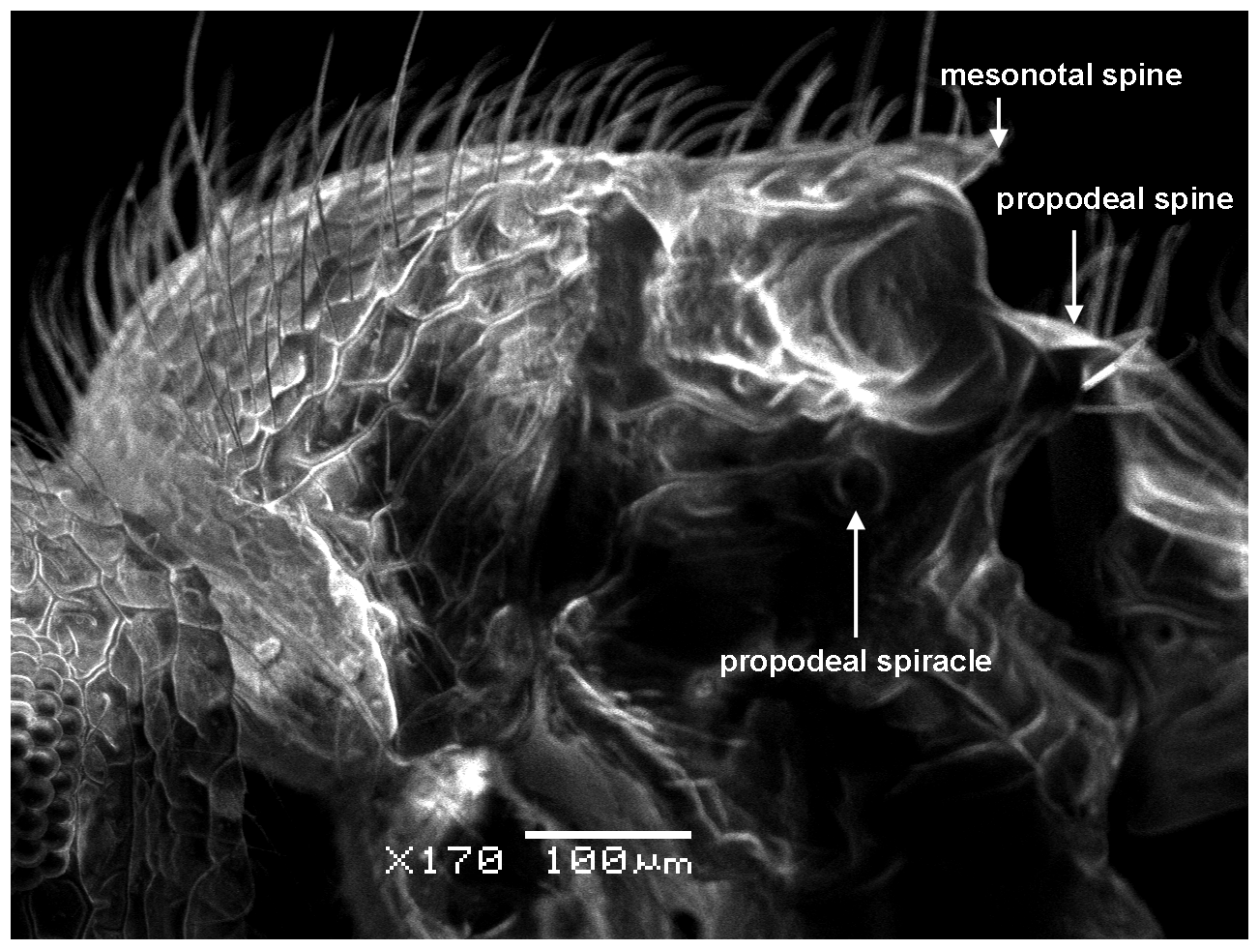
*Meranoplus pulcher* sp. n., paratype worker, mesosoma in profile.

**Figure 6:**
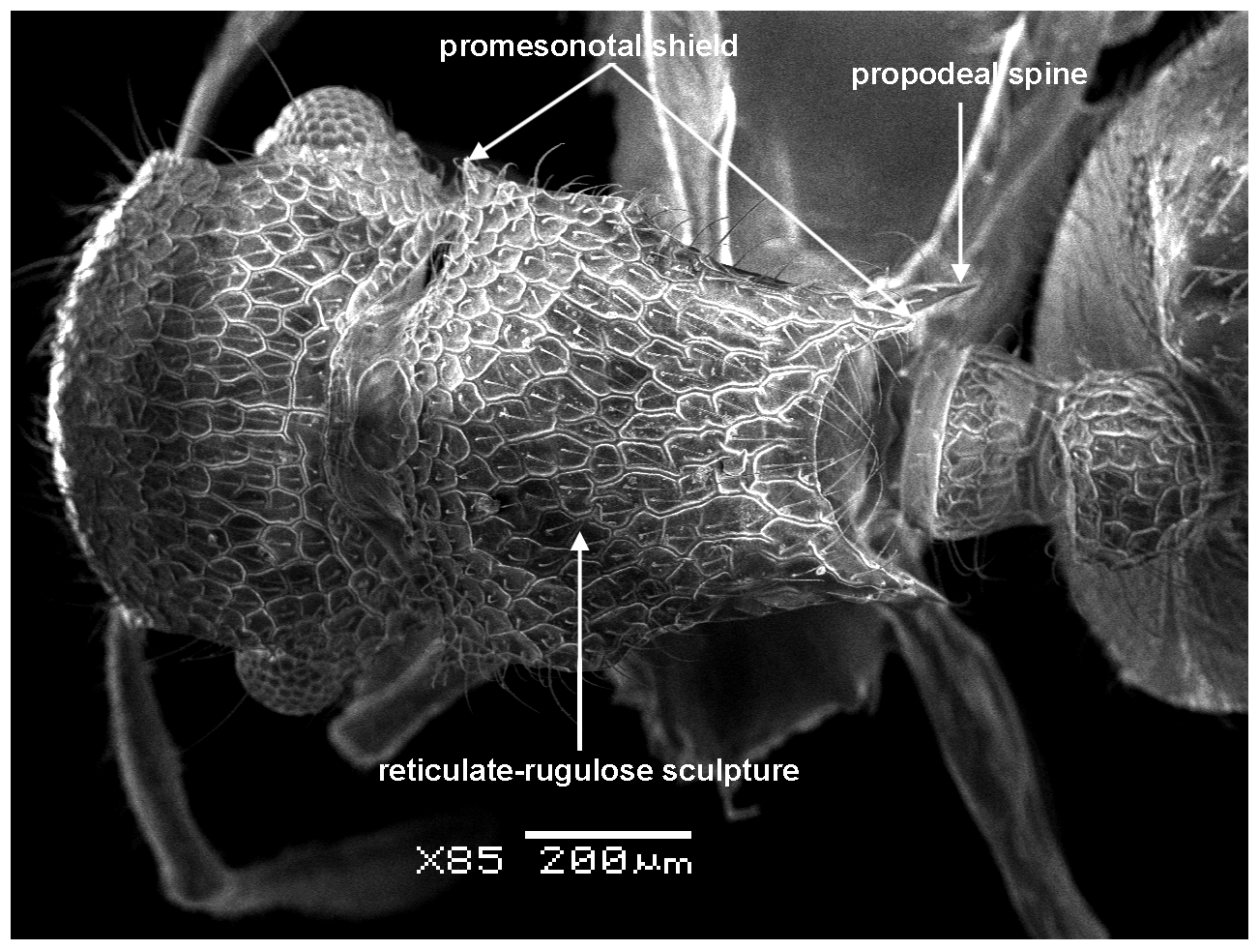
Mesosoma in dorsal view.

**Figure 7:**
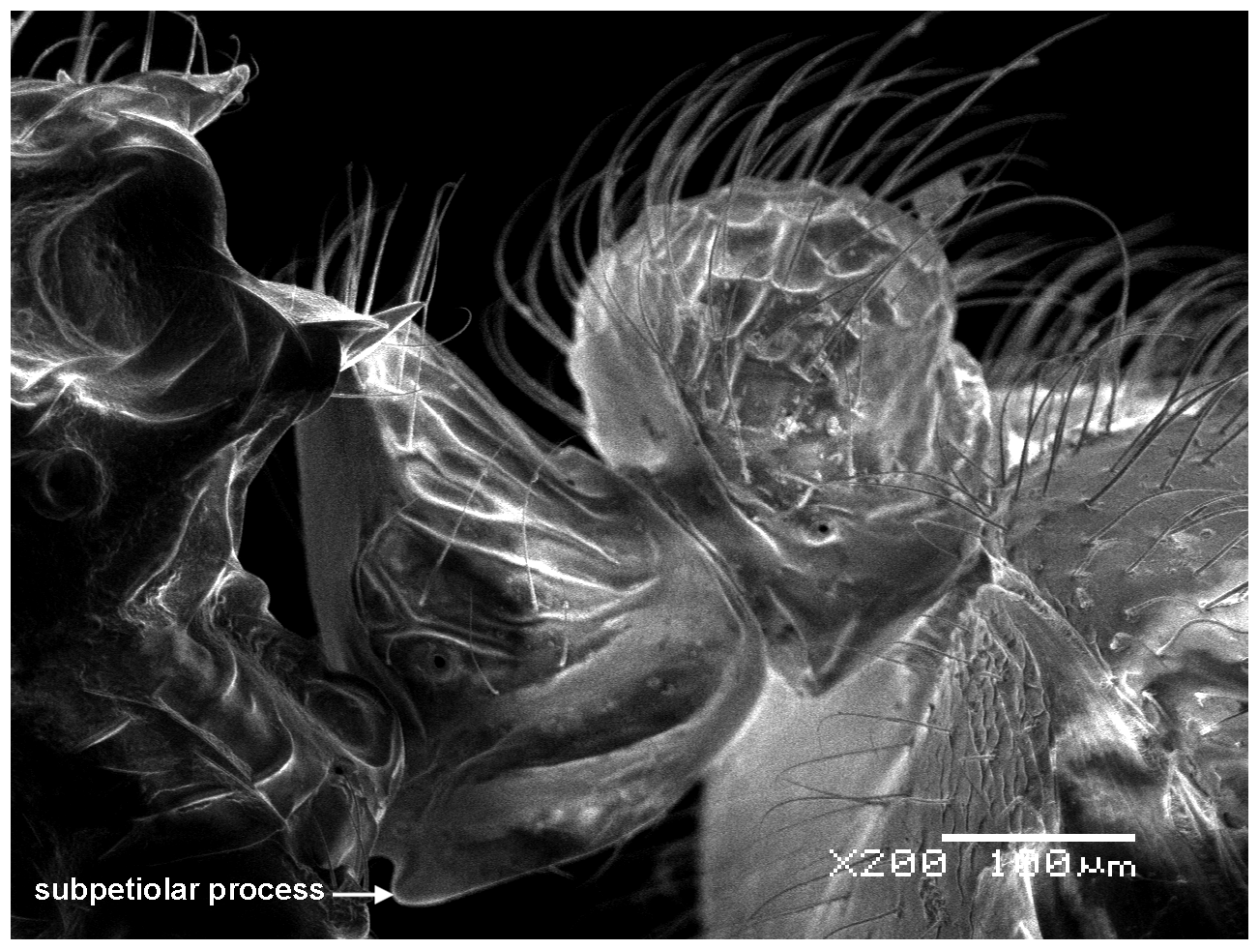
Waist in profile.

**Figure 8:**
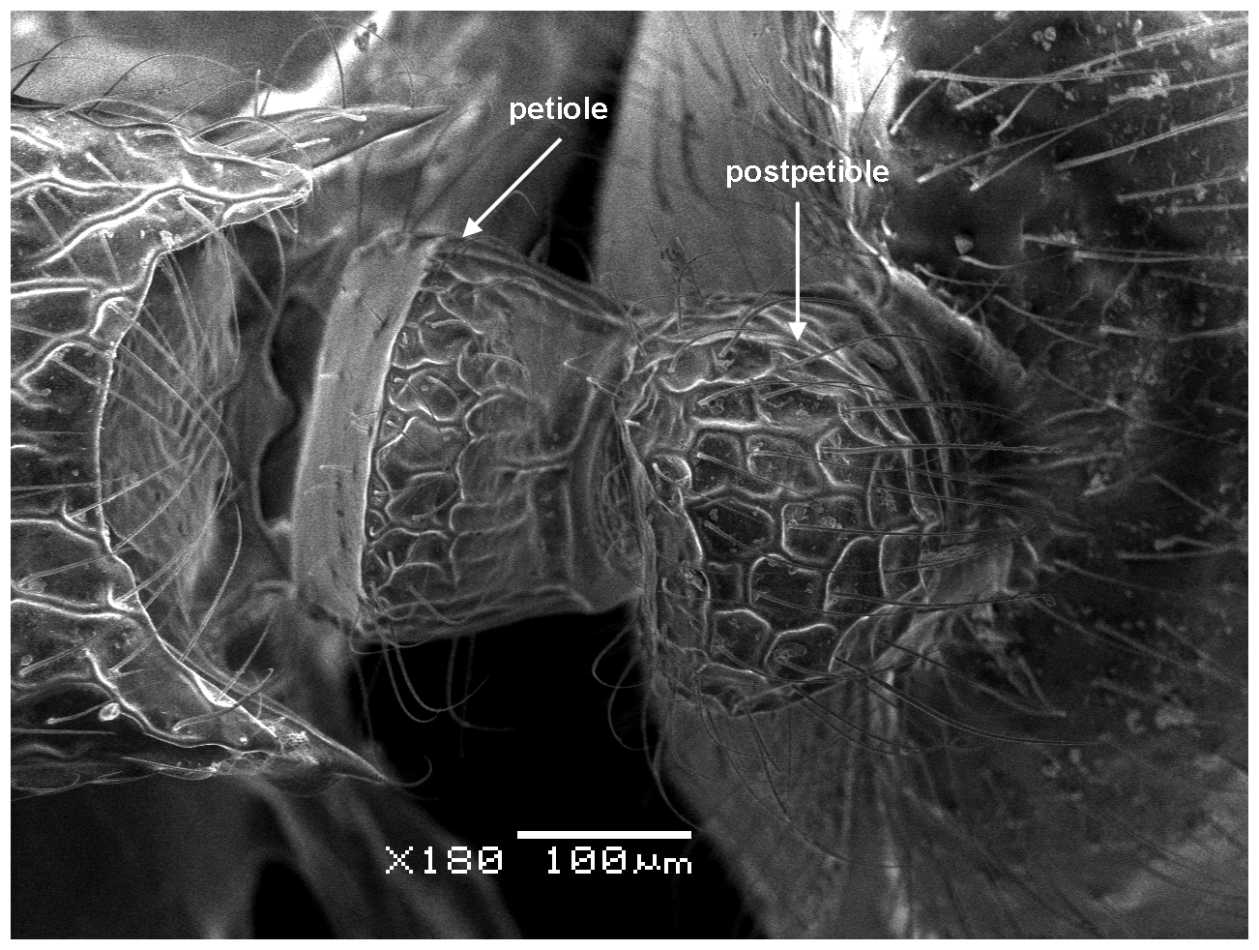
Waist in dorsal view.

**Figure 9:**
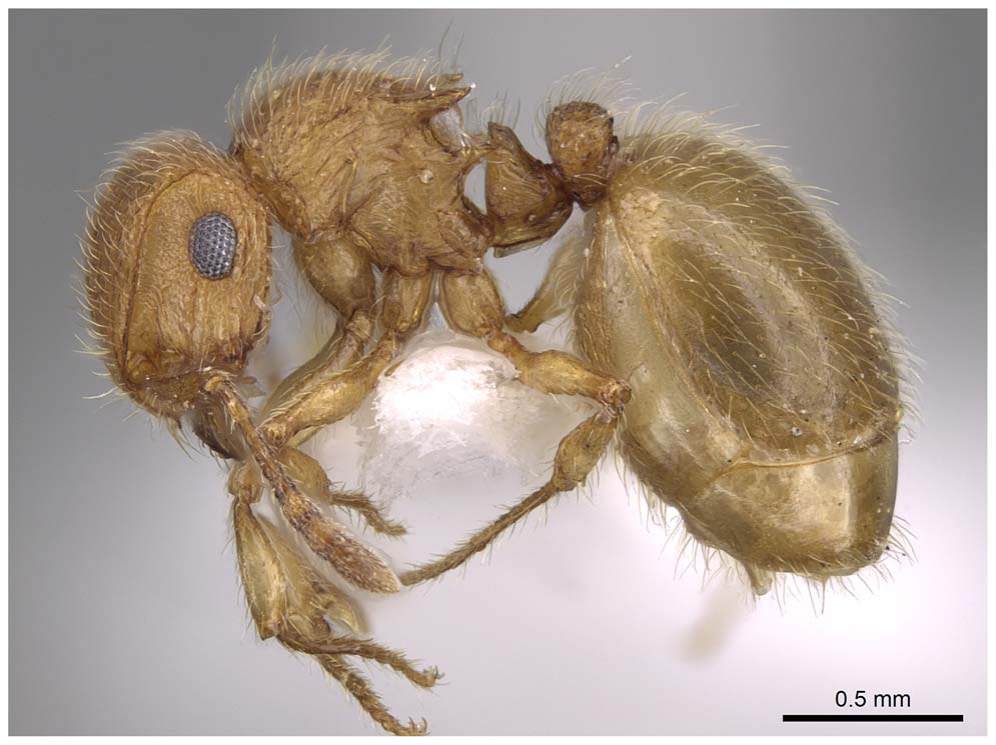
*Meranoplus pulcher* sp. n., paratype worker, body in profile.

**Figure 10:**
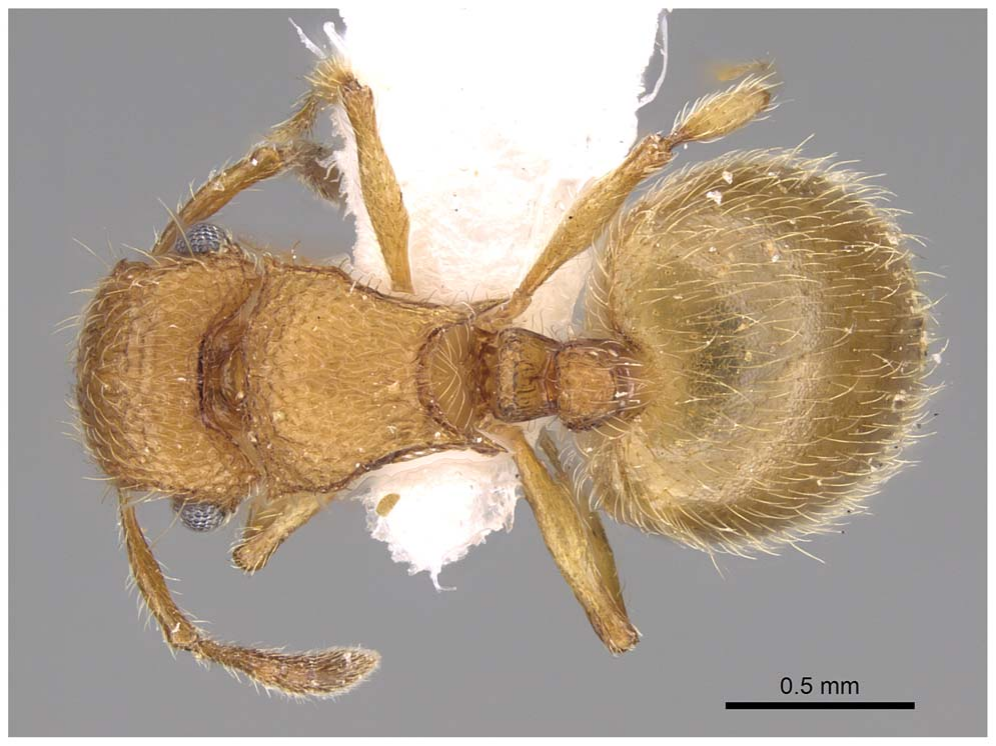
Body in dorsal view.

**Figure 11:**
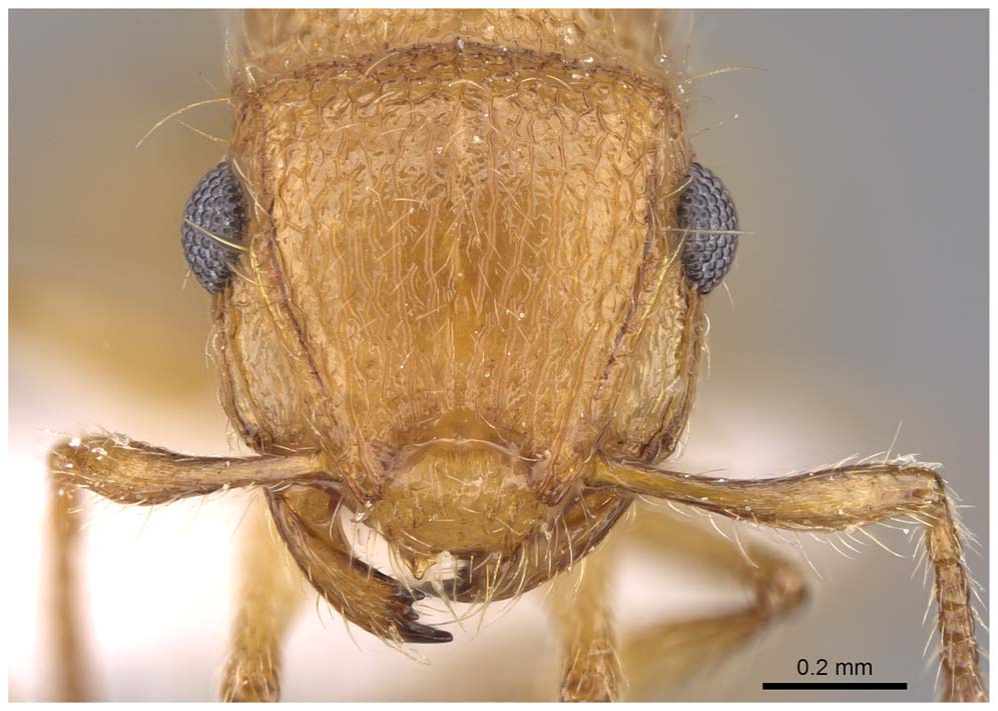
Head in full-face view. CASENT 0914336, Photographer: Michele Esposito, copyright www.antweb.org.

urn:lsid:zoobank.org:act:A5035370-971E-451B-B15E-23F8BBCE5847.

### Holotype worker

SAUDI ARABIA, Al-Baha Province, Shada Al Ala, 19°51.066'N, 41°18.037'E, 1325 m, 23.IV.2014, P. T. *(Al Dhafer et al. Leg.)*, deposited in KSMA, King Saud Museum of Arthropods, College of Food and Agriculture Sciences, King Saud University, Riyadh, Kingdom of Saudi Arabia.

### Paratypes workers

All the following paratype specimens are deposited in KSMA, 3 workers, same locality and data as the holotype; 1 worker with same data as the holotype except the collecting data 15.II.2014; 1 worker, SAUDI ARABIA, Asir Province, Raydah, 18°11.749'N, 42°23.345'E, 1614 m, 28.IV.2014, P.T. *(Al Dhafer et al. Leg.)*, 3 workers, Shada Al Ala, 19°50.575'N, 41°18.691'E, 1666 m, 23.VIII.2014, P. T. *(Al Dhafer et al. Leg.)*; 9 workers, Shada Al Ala, 19°50.411'N, 41°18.686'E, 1611 m, 23.VIII.2014, P. T. *(Al Dhafer et al. Leg.)*; 3 workers, Shada Al Ala, 19°50.329'N, 41°18.604'E, 1563 m, 23.VIII.2014, P. T. *(Al Dhafer et al. Leg.)*; 5 workers, Shada Al Ala, 19°50.710'N, 41°18.267'E, 1474 m, 23.VIII.2014, P. T. *(Al Dhafer et al. Leg.)*; 5 workers, Shada Al Ala, 19°51.066'N, 41°18.037'E, 1325 m, 23.VIII.2014, P. T. *(Al Dhafer et al. Leg.)*; 4 workers, Asir Province, Raydah, 18°11.618'N, 42°23.420'E, 1772 m, 26.VIII.2014, P. T. *(Al Dhafer et al. Leg.)*; 1 workers, Asir Province, Raydah, 18°11.749'N, 42°23.345'E, 1614 m, 26.VIII.2014, P. T. *(Al Dhafer et al. Leg.)*; unique specimen identifier CASENT 0914336, in (CASC) California Academy of Science Collection, San Francisco, California, USA.

### Worker measurements

Maximum and minimum based on all specimens, n = 5, (holotype): TL 3.20-3.70 (3.27), HL 0.77–0.87 (0.80), HW 0.67–0.82 (0.72), HLA 0.25–0.30 (0.25), CW 0.22–0.27 (0.30), CDD 0.12–0.15 (0.12), SL 0.47–0.62 (0.60), EL 0.17–0.22 (0.17), EW 0.12–0.15 (0.15), PML 0.40–0.52 (0.47), PWA 0.62–0.75 (0.70), PWP 0.37–0.47 (0.45), SPL 0.17–0.22 (0.22), WL 0.75–0.87 (0.77), PTL 0.12–0.17 (0.20), PTH 0.30–0.42 (0.37), PPL 0.15–0.22 (0.22), PPH 0.25–0.35 (0.32), ATW 1.02–1.22, (1.12) ATL 0.97–1.15 (1.05), CI 87–94 (90), SI 67–82 (83), OMI 63–80 (68), CDI 0.44–−0.68 (40), SEI 31–43 (28), PMI 144–155 (149), PPI 60–80 (69), PTI 40–46 (54), PWI 82–93 (96), CS 0.72–0.84 (0.76), EYE 38–47 (42) (n = 5).

### Diagnosis

**Figure 12:**
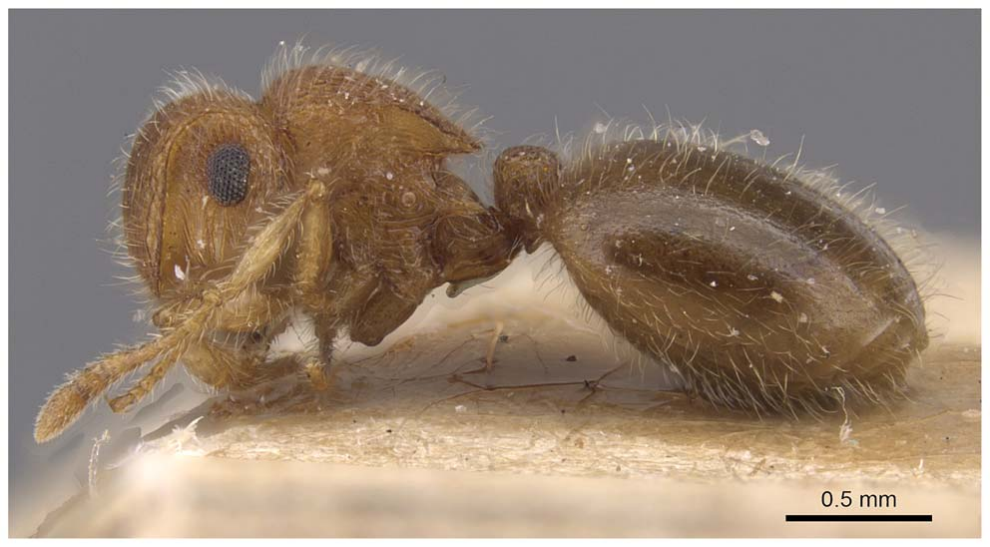
*Meranoplus margettii*, body in profile.

**Figure 13:**
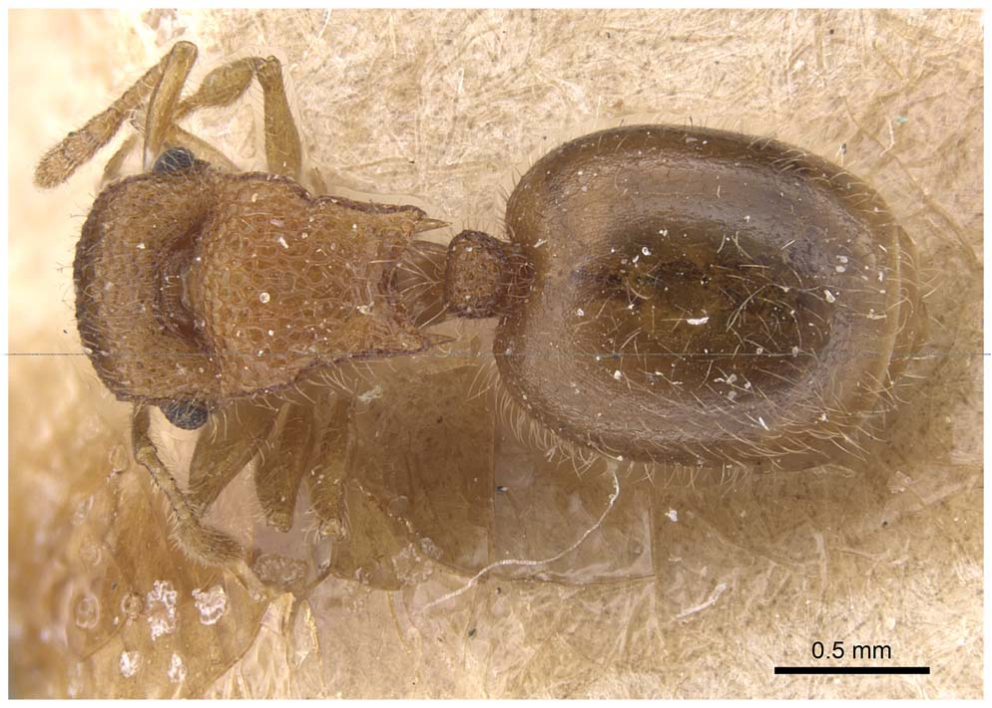
*Meranoplus *margettii**, body in dorsal view.

Although *M. pulcher* sp. n. is superficially similar to *M*. *magrettii* (Figs. 12, 13), it can be readily distinguished by the following contrasting characters: Colour: *M. pulcher* is yellow, *M*. *magrettii* is light to dark brown; anterior clypeal margin: distinctly concave in *M*. *pulcher*, more or less flat to shallowly concave in *M*. *magrettii*; subpetiolar process: in *M*. *pulcher* short and triangular, in *M*. *magrettii* the process is more developed forming a short finger or a less developed process; petiolar sculpture: posterior face of petiolar node areolate-rugose in *M*. *pulcher* and smooth in *M*. *magrettii*; sculpture of first gastral tergite: superficially and finely shagreenate in *M*. *pulcher*, in *M*. *magrettii* the sculpture of first gastral tergite varies from dense shagreenate or reticulate punctate.

### Description

#### Head

Head slightly longer than broad with convex sides and straight posterior margin; anterior clypeal margin distinctly concave with well-developed clypeal carinae; mandibles armed with four teeth; eyes relatively large (EL 0.25–0.26 x HW; EYE 38–47) with 12 ommatidia in the longest row; scapes when laid back from their insertions just reach posterior margin of eyes; scrobal carinae well-developed.

#### Mesosoma

Anterior pronotal corners armed with a pair of short triangular teeth; promesonotal shield distinctly broader than long (PMI 144–155) widening behind pronotum; promesonotal suture absent; posterior corners of mesonotum armed with a pair of sharp spines; posterior mesonotal margin between spines strongly concave and without secondary armament; propodeal spines long and sharp originating at level of propodael spiracles and curved upwards; propodeal lobes well-developed.

#### Waist

Petiole cuneate in profile, sessile, with a broad anterior margin and a narrow acute dorsum; petiolar and postpetiolar anteroventral processes present; postpetiole nodiform, subrectangular in profile, taller than broad (PPI 60–80).

#### Sculpture

Mandibles longitudinally striated; cephalic dorsum densely and finely longitudinally rugulose, posterior margin areolate-rugose; promesonotal shield, posterior face of petiolar node and postpetiole dorsum reticulate rugulose, anterior petiolar face smooth and sides transversally rugulose; first gastral tergite finely and densely shagreenate.

#### Pilosity

All body surface covered with fine, pale, profuse hairs.

#### Colour

Colour unicolorous yellow, in some specimens, postpetiole and posterior margin of first gastral tergite brownish. The six examined specimens showed a clear size variation.

### Etymology

The species name is derived from the Greek word “pulcher” that means “beautiful” referring to the attractive appearance of this ant species.

### Habitat

**Figure 14:**
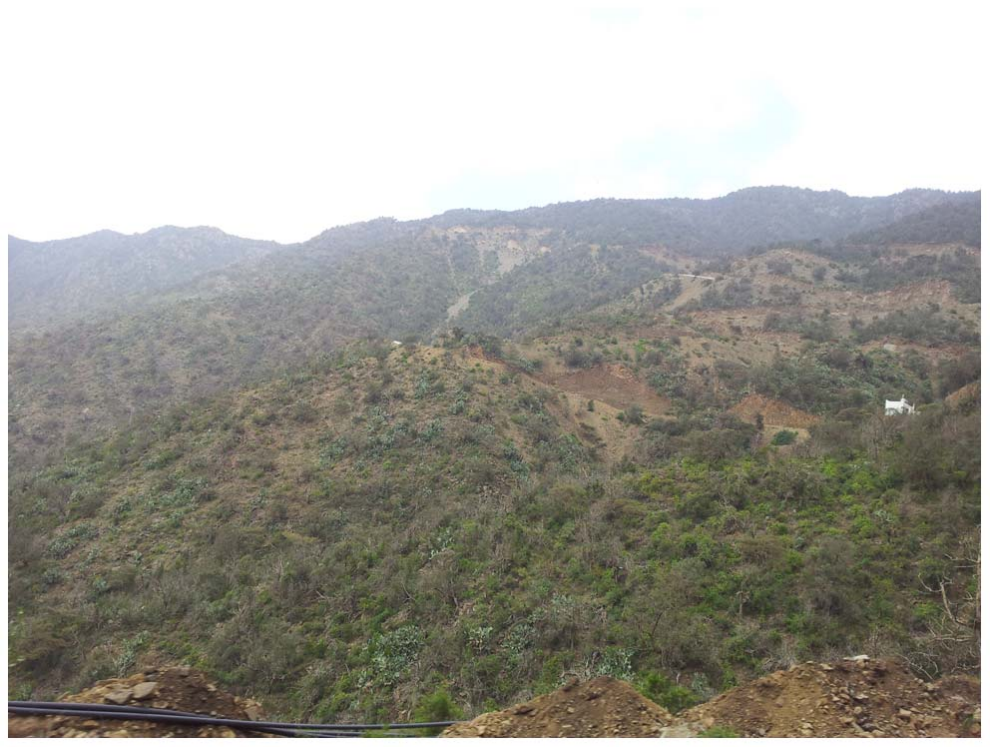
Type locality of *Meranoplus pulcher* sp. n., Shada Al Ala Protectorate, Saudi Arabia.

Twenty five workers of the new species were collected from Al-Baha Province, Shada Al Ala Protectorate (Fig. 14) and six workers from Raydah Protectorate. Both collections were from pitfall traps placed next to *Acacia* trees. The soil was extremely dry with abundant dry seeds of shrubs. Despite several hours of observing the nest no additional specimens were found. Pitfall trapping is apparently an efficient method for collecting this group of ants.

### Key to the Afrotropical species of *Meranoplus magrettii*-group based on workers

In the key to Afrotropical species (Bolton [3]:48), *Meranoplus pulcher* sp. n. will key to couplet 7 along with *peringueyi* and *magrettii*. Couplet 7 is modified here to separate the three species of the *M. magrettii*-group.

7. Mandibles armed with 5 teeth *peringueyi* Emery 

- Mandible armed with 4 teeth 8

**Figure 15:**
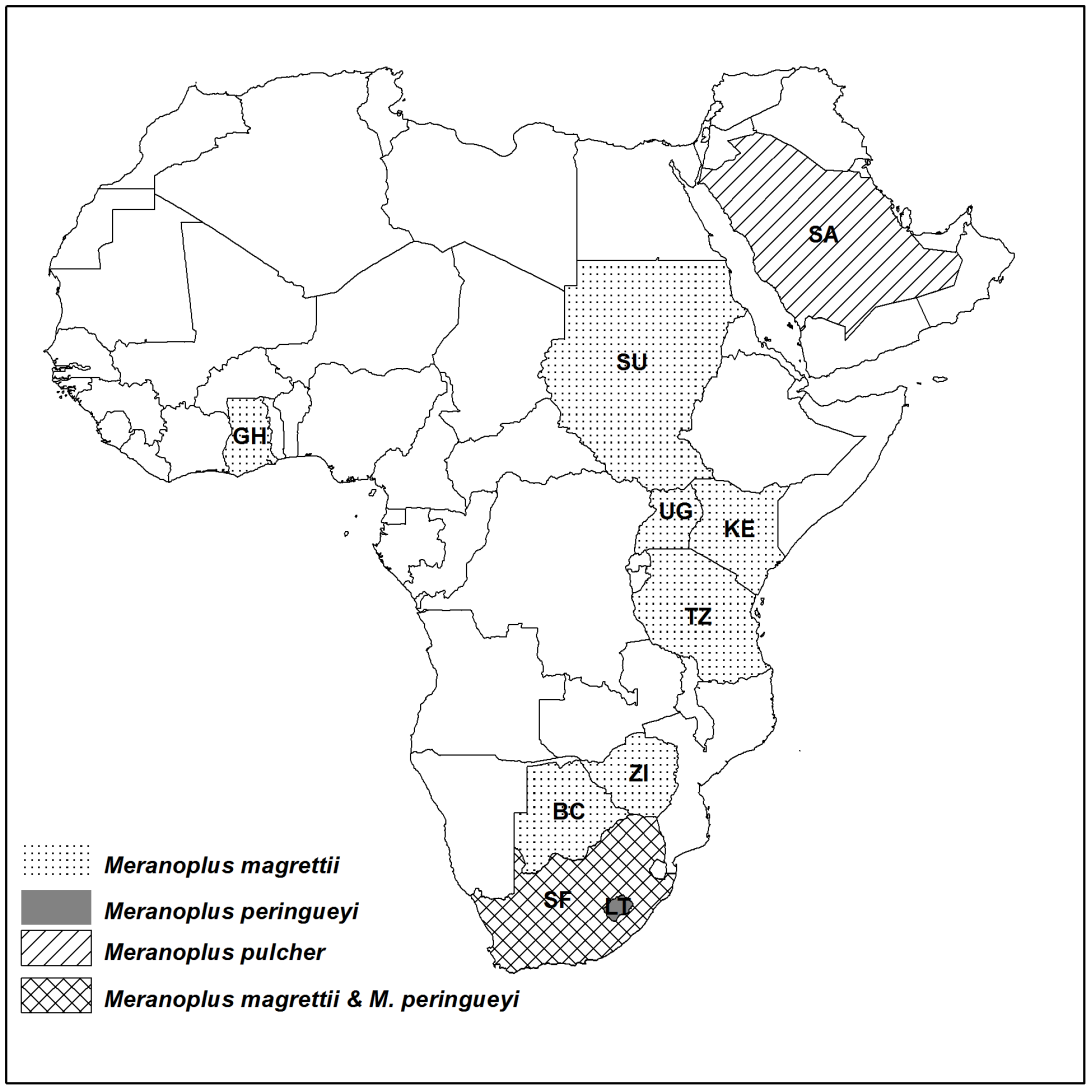
Geographic distribution of species of the *Meranoplus* margettii-group.

8 Colour moderately dark brown, Subpetiolar process more developed, in the form of a short finger (Fig. 12), Posterior face of petiolar node smooth (Fig. 13). (Ghana, Sudan, Uganda, Kenya, Tanzania, Zimbabwe, Botswana, South Africa, Fig. 15) *magrettii* André 

- Colour yellowish, Subpetiolar process present but short (Fig. 7), Posterior face of petiolar node areolate-rugose. (Fig. 8) (Saudi Arabia, Fig. 15) *pulcher* sp. n.

### Zoogeography of the southwestern region of Kingdom of Saudi Arabia

The KSA is located at the interchange of three major biogeographical realms, the Palaearctic, Afrotropical and Oriental. Much mixing of these faunal elements has occurred. Geologically, the Ethiopian and Arabian Peninsula highlands and mountains separated approximately 13 mybp producing the Great Rift Valley through a rifting process as the African continental crust separated [34], [35]. Biogeographically, the southwestern region of the KSA belongs to the Afrotropical region [21]–[23], [25]–[33].

Studies of other taxonomic groups of insects have revealed similar results, for example, the Scythrididae (flower moths) (Lepidoptera: Gelechioidea) were treated for the Palaearctic region [36] and the Afrotropical species *Scythris albocanella* Bengtsson 2002 was recorded from various localities in southwestern region of KSA. In addition, Marnert *et al.* [37] studied the pseudoscorpion arachnids (Pseudoscorpiones) of the region and concluded that the southwestern region of KSA has a clear faunal similarity with the Afrotropical region.

At least ten pantropical ant genera have been recorded from this region of KSA including *Anochetus* Mayr, 1861, *Cryptopone* Emery, 1893, *Cerapachys* Smith, 1857 *Dorylus* Fabricius, 1793, *Hypoponera* Santschi, 1938, *Leptogenys* Roger, 1861, *Melissotarsus* Emery, 1877, *Platythyrea* Roger, 1863, *Polyrhachis* F. Smith, 1857 and *Tetraponera* F. Smith, 1852. Strong biogeographical affinities are with the Afrotropical Region [38]. The sole faunistic work carried out for the knowledge of the ants of KSA [38] recorded 89 species from southwestern region, 18 of which having an Afrotropical distribution (table 1). Despite limited amount of specimens collected from this region mentioned in the above work, preliminary conclusions supported an Afrotropical faunal relationship of the region. Recently, six new species were described from the region [28]–[33], taxa more closely related to Afrotropical congeners (Table 2). An approximate estimate of the relative percentage of the Afrotropical faunal similarity of the region is 31%. Taking into account the unidentified materials accumulated over the last ten years from the region by the authors and also the vast area of the region, some of which has not been surveyed, it is expected this number will increase.

**Table 1:** Ants (Hymenoptera: Formicidae) species recorded from southwestern Kingdom of Saudi Arabia with a known Afrotropical distribution.

Species	Locality in KSA	Date	Type locality
*Anochetus traegaordhi* Mayr, 1904	Fayfa	30.iii.1983	Sudan
*Camponotus empedocles* Emery, 1920	Anamas	18.ix.1983	Zimbabwe
	Wadi Azizah	18.ix.1983	Zimbabwe
	Wadi al Amar	18.ix.1983	Zimbabwe
	Wadi Majarish	22.iii.1982	Zimbabwe
	Tanuma	8.iv.1983	Zimbabwe
*Camponotus ilgii* Forel	Fayfa	28-30.iii.1983	Ethiopia
*Camponotus flavomarginatus* Mayr, 1862	Anamas	8.iv.1983	Ghana
	Sawdah Mountain	9.iv.1983	Ghana
*Camponotus sericeus* (Fabricius, 1798)	Wadi Majarish	7.i.83	Senegal
*Camponotus thales* Forel, 1910	Anamas	8. iv.83	Lesotho
	Sawdah Mountain	9. iv.83	Lesotho
*Cataglyphis abyssinica* (Forel, 1904)	Abu Arish	3.iv.1983	Ethiopia
*Crematogaster senegalensis* Roger, 1863	Sug al Ahad (Asir)	26.iii.83	Senegal
*Crematogaster luctans* Forel, 1907	Fayfa	29.iii.1983	Kenya
	Fayfa	6.iv.2013	Kenya
*Lepisiota incisa* (Forel, 1913)	Anamas	8. iv.83	Democratic Republic of Congo
*Lepisiota obtusa* (Emery, 1901)	Abu Arish	25.iii.83	Ethiopia
*Leptogenys maxillosa* (Smith, 1858)	Fayfa	30.iii.1983	Mauritius
*Melissotarsus emeryi* Forel, 1907	Fayfa	29.iii.1983	Ethiopia
*Monomorium afrum* André, 1884	Abha-Najran RD	10.iv.1983	Sudan
*Monomorium schultzei* Forel, 1910	Wadi Majarish	3.i.1983	South Africa
*Platythyrea modesta* Emery, 1899	Fayfa	30.iii.1983	Cameroun
	Raydah (Asir)	26.viii.2014	Cameroun
*Polyrhachis viscosa* Smith, 1858	Fayfa	31.iii.1983	South Africa
*Strumigenys arnoldi* Forel, 1913	Dhi Ayn (Al Baha)	20.ix.2011	Zimbabwe
*Syllophopsis cryptobium* Santschi, 1921	Wadi Bagara	10.xi.2012	Democratic Republic of Congo
*Tetramorium doriae* Emery, 1881	Wadi Elzaraeb (Al Baha)	15.v.2010	Ethiopia
*Tetramorium khyarum* Bolton, 1980	Dhi Ayn (Al Baha)	23.ix.2011	Nigeria
*Tetramorium sericeiventre* Emery, 1877	Al Tawlah	8.iv.1983	Ethiopia
	Anamas	8.iv.1983	Ethiopia

**Table 2:** Formicidae species described from Kingdom of Saudi Arabia with Afrotropical congeners.

Species	Type Locality	Coordinates	Altitude	Date
*Tetramorium amalae* Sharaf & Aldawood, 2012	Al Bahah, Amadan Forest	20.20000°N 41.21667°E	1881 m	19.V.2010
*Tapinoma wilsoni* Sharaf & Aldawood, 2012	Al Baha, Al Sarawat Mountains, Dhi Ayn	19.92972°N 41.44278°E	741 m	15.v.2011
*Aenictus arabicus* Sharaf & Aldawood, 2012	Al Baha-Mukhwah Aqaba RD	20.00000°N ’41.43758°E	1300 m	19.IV.2012
*Monomorium dryhimi* Sharaf & Aldawood, 2011	Al Bahah province, Amadan forest	20.20000°N 41.21667°E	1881 m	19.V.2010
*Monomorium kondratieffi* Sharaf & Aldawood, 2013	Al Bahah province, AlUrdiya Governorate, Wadi Qonouna	19.42936°N 41.60503°E	353 m	12.v.2011
*M. sarawatensis* Sharaf & Aldawood, 2013	Al Baha-Mukhwah Aqaba RD	20.00000°N ’41.43758°E	1300 m	19.IV.2012

## Discussion

*Meranoplus pulcher* sp. n. is the first member of the genus recorded from KSA and from the vast Arabian Peninsula. Following Bolton [3], it belongs to *M. magrettii*-group and cannot be identified using the available keys to species of Afrotropical [3], Malagasy [5], Oriental [11] or Australian [13], [17] regions. *Meranoplus pulcher* sp. n. is similar to *M. magrettii* André from Sudan to which it will key to in Bolton [3], sharing the following characters: mandibles striate and armed with four teeth, anterior pronotal corners armed with a pair of short triangular teeth, promesonotal shield narrowing behind pronotum, posterior corners of mesonotum armed with a pair of short spines, posterior mesonotal margin concave and unarmed, petiole cuneate in profile and postpetiole nodiform.

The habitats of *M. pulcher* sp. n. and *M. magrettii* apparently are not similar. The latter species is restricted to sub-Saharan Africa and has been collected from savannah, open-woodland and grassland habitats [3]. *Meranoplus pulcher* sp. n.is apparently restricted to juniper woodlands of southwestern mountains of KSA. The author (MRS) has made extensive collections of ants from the southwestern KSA. Typical Afrotropical ant genera mentioned above (e.g. *Strumigenys*, *Anochetus*, *Pachycondyla*, *Cerapachys*, *Dorylus* etc.) were commonly encountered. For example, the Afrotropical species, *S. arnoldi* Forel was reported from Al-Baha Province [39] providing evidence of faunal similarities with the Afrotropical Region. Additional future collections from this area of KSA will no doubt provide further evidence of this biographical connection.

The record of *M. pulcher* sp. n. of the Afrotropical *margettii*-group is an additional evidence of the Afrotropical faunal similarities of the southwestern mountains of KSA which is consistent with other faunal influences for the Region [21]–[23], [25]–[33].
