# Correction: First Record of the Myrmicine Ant Genus *Meranoplus* Smith, 1853 (Hymenoptera: Formicidae) from the Arabian Peninsula with Description of a New Species and Notes on the Zoogeography of Southwestern Kingdom Saudi Arabia

**DOI:** 10.1371/journal.pone.0118389

**Published:** 2015-03-18

**Authors:** The PLOS ONE Staff

There is a word missing in the article title. The correct title is: First Record of the Myrmicine Ant Genus *Meranoplus* Smith, 1853 (Hymenoptera: Formicidae) from the Arabian Peninsula with Description of a New Species and Notes on the Zoogeography of Southwestern Kingdom of Saudi Arabia. The correct citation is: Sharaf MR, Al Dhafer HM, Aldawood AS (2014) First Record of the Myrmicine Ant Genus *Meranoplus* Smith, 1853 (Hymenoptera: Formicidae) from the Arabian Peninsula with Description of a New Species and Notes on the Zoogeography of Southwestern Kingdom of Saudi Arabia. PLoS ONE 9(11): e111298. doi:10.1371/journal.pone.0111298

## References

[1] Sharaf MR, Al Dhafer HM, Aldawood AS (2014) First Record of the Myrmicine Ant Genus *Meranoplus* Smith, 1853 (Hymenoptera: Formicidae) from the Arabian Peninsula with Description of a New Species and Notes on the Zoogeography of Southwestern Kingdom Saudi Arabia. PLoS ONE 9(11): e111298 doi:10.1371/journal.pone.0111298 25375104 10.1371/journal.pone.0111298 PMC4222904

